# The rat rotenone model reproduces the abnormal pattern of central catecholamine metabolism found in Parkinson's disease

**DOI:** 10.1242/dmm.049082

**Published:** 2022-01-24

**Authors:** Regev Landau, Reut Halperin, Patti Sullivan, Zion Zibly, Avshalom Leibowitz, David S. Goldstein, Yehonatan Sharabi

**Affiliations:** 1 Neuroautonomic Service, Chaim Sheba Medical Center, Affiliated with the Tel Aviv University Sackler Faculty of Medicine, Tel-HaShomer 5265601 Ramat Gan, Israel; 2 Autonomic Medicine Section, Clinical Neurosciences Program, Division of Intramural Research, National Institute of Neurological Disorders and Stroke, National Institutes of Health, Bethesda, MD 20892-0151, USA; 3 Department of Neurosurgery, Chaim Sheba Medical Center, Affiliated with the Tel Aviv University Sackler Faculty of Medicine, Tel-HaShomer 5265601 Ramat Gan, Israel

**Keywords:** Rotenone, Parkinson's disease, DOPAL, Vesicular uptake, Aldehyde dehydrogenase, Dopamine, Norepinephrine, Catecholamine, Catechol, Lewy body diseases

## Abstract

Recent reports indicate that Parkinson's disease (PD) involves specific functional abnormalities in residual neurons – decreased vesicular sequestration of cytoplasmic catecholamines via the vesicular monoamine transporter (VMAT) and decreased aldehyde dehydrogenase (ALDH) activity. This double hit builds up the autotoxic metabolite 3,4-dihydroxyphenylacetaldehyde (DOPAL), the focus of the catecholaldehyde hypothesis for the pathogenesis of PD. An animal model is needed that reproduces this abnormal catecholamine neurochemical pattern. Adult rats received subcutaneous vehicle or the mitochondrial complex 1 inhibitor rotenone (2 mg/kg/day via a minipump) for 10 days. Locomotor activity was recorded, and striatal tissue sampled for catechol contents and catechol ratios that indicate the above abnormalities. Compared to vehicle, rotenone reduced locomotor activity (*P*=0.002), decreased tissue dopamine concentrations (*P*=0.00001), reduced indices of vesicular sequestration (3,4-dihydroxyphenylacetic acid (DOPAC)/dopamine) and ALDH activity (DOPAC/DOPAL) (*P*=0.0025, *P*=0.036), and increased DOPAL levels (*P*=0.04). The rat rotenone model involves functional abnormalities in catecholaminergic neurons that replicate the pattern found in PD putamen. These include a vesicular storage defect, decreased ALDH activity and DOPAL build-up. The rat rotenone model provides a suitable *in vivo* platform for studying the catecholaldehyde hypothesis.

## Abstract (precis)

**Summary:** This study presents an animal model that reflects the neurochemical pattern found in Parkinson's patients, the basis of the new and evolving catecholaldehyde hypothesis for the disease.

## INTRODUCTION

**Fig. 1.**
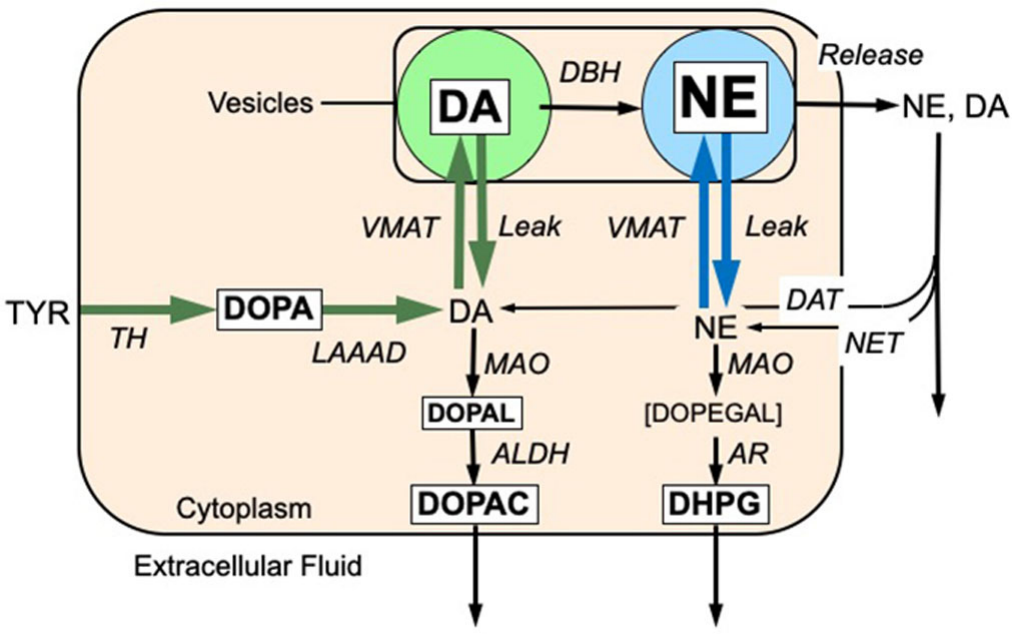
**Concept diagram showing enzymatic steps in the synthesis, vesicular storage, release, reuptake and metabolism of dopamine (DA) and norepinephrine (NE).** The six endogenous catechols (in white rectangles) were measured simultaneously. DA is synthesized in the neuronal cytoplasmic via tyrosine hydroxylase (TH) acting on tyrosine to form 3,4-dihydroxyphenylalanine (DOPA) and then L-aromatic-amino-acid decarboxylase (LAAAD) acting on DOPA. Most of cytoplasmic DA is taken up into vesicles via the vesicular monoamine transporter (VMAT) but a minority undergoes enzymatic oxidation catalyzed by monoamine oxidase (MAO) to form 3,4-dihydroxyphenylacetaldehyde (DOPAL). DOPAL is metabolized by aldehyde dehydrogenase (ALDH) to form 3,4-dihydroxyphenylacetic acid (DOPAC), which exits the cell. DA in the vesicles undergoes enzymatic hydroxylation by DA-beta-hydroxylase (DBH) to form NE. Catecholamines released into the extracellular fluid is taken back up into the cytoplasm via the cell membrane DA transporter (DAT) or NE transporter (NET). NE in the cytoplasm can undergo vesicular uptake or MAO-catalyzed oxidative deamination to form 3,4-dihydroxyphenylglycolaldehyde (DOPEGAL), which is reduced by aldehyde/aldose reductase (AR) to form 3,4-dihydroxyphenylglycol (DHPG). DHPG rapidly exits the neuron. Font sizes correspond roughly to tissue concentrations of the analytes in rat striatum.

Parkinson's disease (PD) is the second most-common aging-related neurodegenerative disease. The movement disorder in PD is known to result from profound striatal dopamine deficiency. The extent of catecholamine depletion in PD, however, is far greater than can be accounted for by loss of innervation alone (Goldstein et al., 2017). This discrepancy suggests that there are populations of dysfunctional catecholaminergic neurons that are ‘sick-but-not-dead’ and, therefore, salvageable (Goldstein et al., 2019). The reason(s) for the selective vulnerability of dopaminergic neurons are incompletely understood. Studies have pointed to a double hit of reduced sequestration of cytoplasmic dopamine into vesicles via the vesicular monoamine transporter (VMAT) and reduced activity of aldehyde dehydrogenase (ALDH) (Fig. 1), which together result in accumulation of the autotoxic catecholaldehyde 3,4-dihydroxyphenylacetaldehyde (DOPAL). DOPAL is the centerpiece of the catecholaldehyde hypothesis for the pathogenesis of PD (Masato et al., 2019). The catecholaldehyde not only is neurotoxic itself but also interacts strongly with intracellular proteins, including alpha-synuclein (Jinsmaa et al., 2020; Burke et al., 2008), a key component in Lewy bodies and a focus of current research about PD pathogenetic mechanisms.

Post-mortem neurochemical analyses of putamen tissue from PD patients have supported the catecholaldehyde paradigm by documenting the double hit and DOPAL accumulation The mean DOPAL/dopamine ratio in PD is about five times that in controls, and DOPAL build-up is determined by decreased vesicular sequestration and decreased ALDH activity (Goldstein et al., 2013). In humans, neuroimaging kinetics studies using ^18^F-DOPA positron emission tomographic scanning showed increased wash out of the tracer, reflecting reduced VMAT activity (Goldstein et al., 2008), and cerebrospinal fluid catechol analyses revealed decreased 3,4-dihydroxyphenylacetic acid levels without concurrent decrease in 5-S-cysteinyldopamine (Goldstein et al., 2016), consistent with reduced ALDH activity. These findings provide *in vivo* support for the occurrence of the double hit in patients with PD.

If the deleterious neurochemical abnormalities resulting from the double hit can be modulated, progression of the neurodegenerative process might be slowed down. Therefore, translational research based on the catecholaldehyde hypothesis requires an animal model that replicates the distinctive neurochemical pattern found in PD.

Parts of the double hit have been used in PD mouse genetic models of low VMAT activity (Miller et al., 2001; Bohnen et al., 2006; Miller et al., 1999; Okamura et al., 2010; Tong et al., 2011) or ALDH knockout mice (Wey et al., 2012; Galter et al., 2003; Grünblatt et al., 2010; Mandel et al., 2005; Molochnikov et al., 2012; Werner et al., 2008), but no animal model demonstrating DOPAL accumulation concurrent with evidence for the double hit has been reported to date.

We chose the rotenone rat model for this purpose. Rotenone is a mitochondrial complex 1 inhibitor that, in rat pheochromocytoma PC-12 cells, produces both components of the double hit and increases endogenous DOPAL levels (Goldstein et al., 2015). Moreover, DOPAL contributes to rotenone-induced cytotoxicity in this model (Lamensdorf et al., 2000). Whether rotenone produces this pattern *in vivo* is unknown. The rat rotenone model is a well-established and characterized animal model of PD (Betarbet et al., 2000; Cannon et al., 2009). Rotenone produces motor dysfunction without extensive destruction of dopaminergic neurons, as documented by detailed neuropathological data in this model (Cannon et al., 2009). The occurrence of neurobehavioral abnormalities unaccounted for by extensive neuronal loss fits with the sick-but-not-dead phenomenon (Goldstein et al., 2019).

The primary objective of this study was to evaluate the central catecholaminergic profile in the rat rotenone model. We also included representative locomotor tests to ascertain that the model replicates the PD-like locomotor abnormalities that have been reported previously. We did not perform neuropathologic analysis in the experimental design since this has already been done in detail (Cannon et al., 2009).

In this study, rats received rotenone or sham treatment via subcutaneous reservoir minipumps. Based on the concepts depicted in Fig. 1, we hypothesized that rotenone builds up striatal DOPAL with respect to dopamine, and decreases neurochemical indices values of vesicular sequestration and ALDH activity. If there is decreased vesicular sequestration of cytoplasmic catecholamines, tissue dopamine and norepinephrine would be depleted with respect to their deaminated metabolites DOPAL, 3,4-dihydroxyphenylglycol (DHPG), and 3,4-dihydroxyphenylacetic acid (DOPAC). If there is decreased ALDH activity, ratios of DOPAC/DOPAL would be decreased.

Regarding the locomotor assessments, we chose tests known to represent the movement abnormalities in this rat model. The tests we performed are described in the original studies that introduced the model (Betarbet et al., 2000; Cannon et al., 2009) .The cylinder test (rearing test) and open-field test tests are well established (Hua et al., 2002; Rial et al., 2014; Schaar et al., 2010; Su et al., 2018), and allow evaluation of the locomotor abnormalities in this model.

## RESULTS

### Motor function measures

**Fig. 2.**
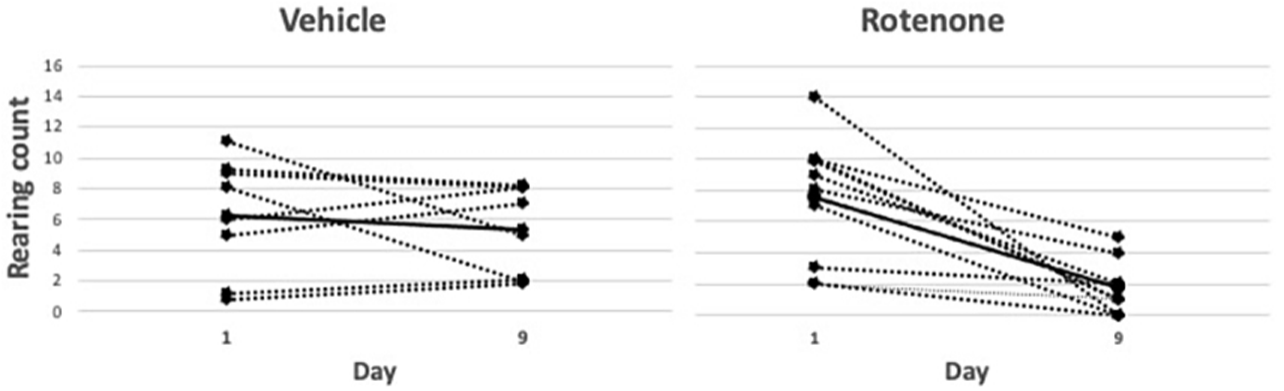
**Comparison of rearing behavior (counts/2 min) between days 1 and 9 in rotenone- and vehicle-treated rats.** Representation of individual animal data (dotted lines) with mean values per group shown as solid lines. There is a significant difference (*t*-test) in rearing count between days in the rotenone group (*P*=0.0015; *n*=8) but not in the vehicle group (*P*=0.41; *n*=11).

**Fig. 3.**
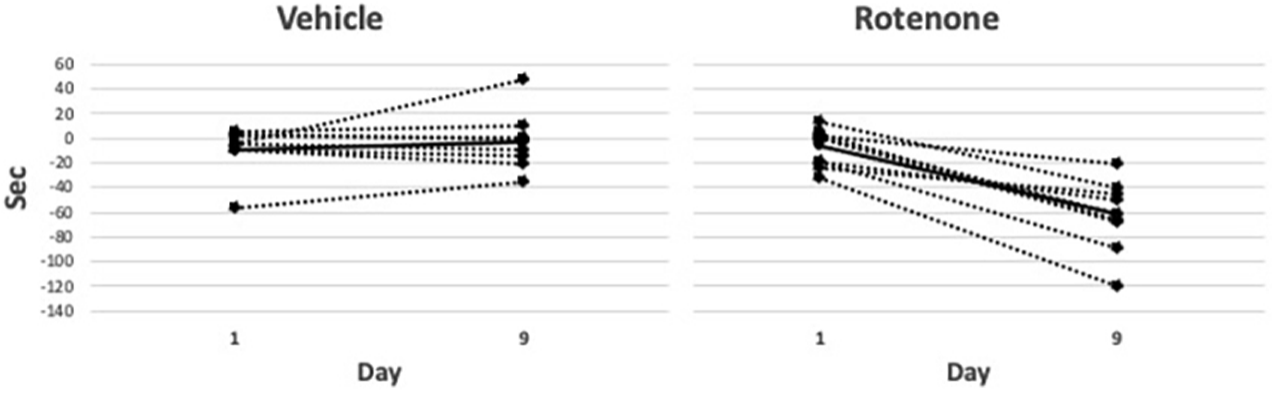
**Activity difference (activity in seconds on day 0 minus activity on day 1 and day 9) in the rotenone- and vehicle-treated groups.** Representation of individual animal data (dotted lines) with mean values per group shown as solid lines. There is a significant difference (*t*-test) in activity, i.e. activity (in s) on day 0 minus activity on days 1 and 9, between days in the rotenone group (*P*=0.0001; *n*=8) but not in the vehicle group (*P*=0.41; *n*=11).

Rotenone exerted clear effects on the locomotor indices, as follows. At baseline and on day 1 there were no differences in the number of rearings/2 min between vehicle- and rotenone-treated groups (baseline: 7.3±1.1 for vehicle, 9.7±0.8 for rotenone, *P*=0.17; day 1: 6.2±1.3, and 7.6±1.1, *P*=0.66), whereas on day 9 the rotenone-treated group reared significantly fewer times than did the vehicle-treated group (1.8±0.5 versus 5.2±1.0; *P*=0.003) (Fig. 2). The groups did not differ in the change in activity time on day 1 (-6.6±4.4 versus −9.1±7.0, *P*=0.92), whereas on day 9 the rotenone-treated group had significantly reduced activity compared to the vehicle-treated group (−62.3±8.8 and −3.0±8.8 s, *P*=0.002) (Fig. 3).

### Neurochemical measures

Rotenone evoked several clear effects on catechol tissue levels. Compared to the vehicle-treated group, the rotenone-treated group had an 82% reduction in tissue dopamine levels (*P*=0.0001); its levels of deaminated metabolites, however, were increased – 1.69-fold for DOPAL (*P*=0.04) and 1.34-fold for DHPG (*P*=0.04) (Table 1). Levels of the DA precursor DOPA increased 2.68-fold (*P*=0.0005). Levels of tissue norepinephrine or DOPAC did not differ between groups (Table 1). The mean DA/DOPA ratio, an index of LAAAD, was also decreased in the rotenone-treated group (*P*=0.0005) (Table 2). Compared to the vehicle-treated group, indices of VMAT activity were all significantly decreased in the rotenone-treated group, i.e. the mean ratio of DA to the sum of its deaminated metabolites (*P*=0.0003), DA/DOPAL ratio (*P*=0.0025), DA/DOPAC ratio (*P*=0.035) and NE/DHPG ratio (*P*=0.012) (Table 2). The mean DOPAC/DOPAL ratio, an index of ALDH activity, was decreased in the rotenone-treated group (*P*=0.036) (Table 2).

**Table 1.**
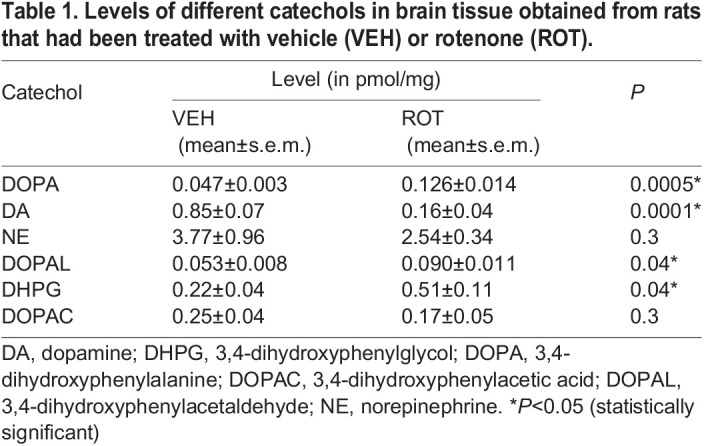
Levels of different catechols in brain tissue obtained from rats that had been treated with vehicle (VEH) or rotenone (ROT).

**Table 2.**
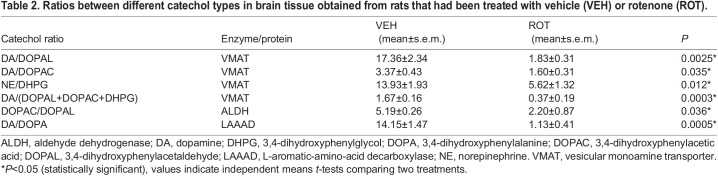
Ratios between different catechol types in brain tissue obtained from rats that had been treated with vehicle (VEH) or rotenone (ROT).

## DISCUSSION

According to the catecholaldehyde hypothesis, a double hit of reduced vesicular sequestration of cytoplasmic dopamine and reduced ALDH activity results in the accumulation of endogenous DOPAL, with multiple deleterious downstream effects. Tissue neurochemistry and neuroimaging data from patients with PD show a distinctive catecholaminergic pattern that fits with the catecholaldehyde hypothesis. Here, we report that systemic administration of rotenone to rats depletes dopamine levels and decreases values for indices of vesicular sequestration and ALDH activity, while increasing DOPAL levels. This is the first report of an animal model that does so. The body of evidence about DOPAL toxicity is based on *in vitro* studies – hence the usefulness of an *in vivo* model that reproduces DOPAL accumulation as well as the double hit.

DOPAL is gaining attention as a factor in neuronal toxicity in general and in particular through its interactions with alpha-synuclein, a central theme in PD pathogenesis (Masato et al., 2019). *In vitro* experiments show that DOPAL oligomerizes, aggregates and forms quinone-protein adducts with alpha-synuclein, likely to be converting the protein to toxic forms. DOPAL directly induces toxic effects on mitochondrial functions, and DOPAL-induced alpha-synuclein oligomers impede synaptic vesicular functions (Plotegher et al., 2017), potentially setting the stage for pathogenic vicious cycles.

It is important for this emerging field of neurochemistry-oriented research to establish an *in vivo* model that demonstrates both the catecholaminergic and neurobehavioral phenotypic features of the disease. There are several animal models for PD. Genetics-based approaches include the generation of transgenic animals and altered gene expression via viral vector is used to highlight genetic aspects of PD. Some models are based on catecholaminergic neurotoxicity evoked by chemical compounds, such as 1-methyl-4-phenyl-1,2,3,6-tetrahydropyridine (MPTP) and 6-hydroxydopamine, which produce both dopamine depletion and parkinsonian locomotor abnormalities. Some models induce profound dopamine depletion by direct destruction of the striatum. The rotenone model presented here, however, is particularly suitable for applying the catecholadehyde hypothesis, according to which the dopamine depletion and neurobehavioral phenotype are related to abnormal catecholamine metabolism in extant neurons. This model is not associated with extensive neuronal destruction.

It is well established that systemic rotenone administration evokes an ‘energy crisis’ by depleting the brain of dopamine. The issue at hand is the link between these abnormalities. Rotenone induces metabolic stress in all cells of the body. But how does it produce a relatively specific syndrome that involves dopamine depletion and, consequently, a parkinsonian movement disorder? According to the catecholaldehyde hypothesis, DOPAL is the missing link. Inhibition of the mitochondrial complex 1 by rotenone decreases availability of NAD+, a required co-factor for detoxifying DOPAL. The energy crisis in dopaminergic neurons also impedes ATP-dependent vesicular uptake of catecholamines, meaning that the fate of cytoplasmic dopamine is shifted towards the formation of DOPAL. Here, we provide key evidence to support such a link, i.e. that there is, indeed, a built up of DOPAL in the rat rotenone model. Since this model features the same distinctive central catecholaminergic pattern that is found post-mortem in PD, the model is suitable for testing the catecholadehyde hypothesis.

Our study assessed effects of rotenone on processes and enzymes by calculating ratios between tissue product and substrate. The assessment of ALDH is straightforward since the direct substrate and immediate product are within the same cellular compartment, the cytoplasm. The assessment of VMAT2 is more complex because multiple metabolites and different intra-neuronal compartments are involved. We used several ratios to assess the effect of rotenone on VMAT2 in both dopaminergic and noradrenergic pathways. All indices point to rotenone-induced inhibition of VMAT2.

There are alternative methods to assess VMAT2 function and ALDH activity that are more direct than measuring catechol ratios. We chose the same methodology we used when working with human tissue: the substrate/product ratios provide indirect measures of these processes. At this point, we thought it important to replicate the abnormal pattern of tissue catechols found post-mortem in PD. A limitation of our approach is that tissue catechol ratios provide only indirect indices of intra-neuronal processes, such as vesicular uptake. A variety of methods are available to examine VMAT2 function, such as uptake and retention of ^3^H-DA. It should be noted that these do not easily separate attenuated active uptake from augmented passive vesicular leakage as determinants of decreased vesicular catecholamine stores. Recent evidence indicates that both types of functional abnormality are found in catecholaminergic neurons in Lewy body diseases (Goldstein et al., 2019).

The finding of increased tissue DOPA levels can be explained by inhibition of LAAAD, which is part of the pattern of metabolic abnormalities found in post-mortem studies (Goldstein et al., 2017). Addressing these complex patterns seems to require a systems biology approach. We have recently reported that computational modeling in patients with Lewy body diseases reveals multiple functional abnormalities in catecholaminergic neurons (Goldstein et al., 2019). The data regarding LAAAD and vesicular sequestration in this present study are in line with the results reported for patients with Lewy body diseases.

In a previous study, using the PC12 cellular model, we found that, as a result of inhibition of complex 1 by rotenone, the suppressive effect of rotenone on ALDH is mediated by decreased availability of the co-factor NAD+ (Goldstein et al., 2015). As for the effect on VMAT2, Sai et al. showed that rotenone downregulates expression of VMAT2 in the PC12 model (Sai et al., 2008). The effect of rotenone on VMAT2 has also been evaluated in the SH-SY5Y cellular model by Watabe and Nakaki, who demonstrated a direct effect of rotenone on the transporter (Watabe and Nakaki, 2008). Moreover, animal studies applying genetic manipulations that were expected to increase DOPAL in dopaminergic neurons show features resembling those in PD (Chiu et al., 2015; Kao et al., 2020; Landrock et al., 2018; Wey et al., 2012). Here, we report that rotenone not only increases DOPAL levels but also produces other catecholaminergic abnormalities that seem relevant to PD pathogenesis; hence the value of this model. Regarding neuropathology, a report by Cannon et al. provided histological evidence of dysfunctional dopaminergic terminals in the rat rotenone model, without severe neuronal loss (Cannon et al., 2009); our neurochemical data fit well with the histological findings of that study.

DA, DOPAL and DOPAC are substrates for catechol-O-methyltransferase (COMT); however, catecholaminergic neurons have little if any COMT activity. This is one reason why measuring tissue DA/DOPAC ratios has long been an accepted method for assessing the turnover of endogenous DA. The tissue turnover of catecholamines depends mainly on the balance of vesicular uptake versus vesicular leakage (Eisenhofer et al., 2004). It is reasonable to infer that the abnormalities in tissue catechol ratios reported here are accounted for by decreased vesicular storage. To our knowledge, there is no simple test of vesicular uptake that can be applied *in vivo*. This is because (1) the concurrent high rate of vesicular leakage, (2) the series arrangement of cellular uptake followed by vesicular uptake of exogenous tracer-labelled catecholamines and, (3) the likelihood of substantial protein reactivity of oxidized catecholamines. Particularly – because of points 1 and 2 – one cannot draw inferences regarding vesicular uptake based on assessing the kinetics of tissue ^3^H-DA. Even after taking cellular uptake into account, it would be difficult, if not impossible, to separate decreased vesicular uptake from increased vesicular permeability when explaining decreased ^3^H-DA tissue levels. Analyzing these processes separately seems to require highly non-physiologic experiments on isolated vesicles. Regarding point 3, when tissue levels of ^3^H-catecholamines were tracked after administration into animals, a substantial proportion of the ^3^H was not accounted for by free levels of all the known catecholamines and their metabolites (Chang et al., 1990). We, therefore, speculate that ^3^H-DOPAL formed in the neuronal cytoplasm oxidizes spontaneously to ^3^H-DOPAL-quinone that, in turn, binds covalently to – i.e. it ‘quinonizes’ – numerous intracellular proteins (Jinsmaa et al., 2018, 2020; Zhu et al., 2004).

The aldose reductase product acting on DOPAL is 3,4-dihydroxyphenylethanol (DOPET), which we included in our assay. As expected, the amount of DOPET detected was small compared to that of DOPAC, i.e. the product of ALDH acting on DOPAL. Directly assaying ALDH activity does not provide the information sought, since it involves supplying NAD co-factor for the assay. If rotenone decreases ALDH activity indirectly via the decreased production of NAD+ resulting from inhibition of complex 1 – which is likely to be the case (Goldstein et al., 2015) – ALDH activity measured in a test tube experiment would not identify this decrease in enzyme activity as a result of rotenone.

Together with the primary objective of this study, i.e. to obtain neurochemical data, we conducted neurobehavioral assessments to link the neurochemical results with motor dysfunction in this model. We used motor function tests known to identify the movement abnormalities in the rotenone model, i.e. the two main tests we used were chosen from the original studies that introduced the rotenone model (Betarbet et al., 2000; Cannon et al., 2009), and in which the full description of the locomotor abnormalities induced by rotenone are described. Our results confirmed the reduced motor mobility and activity following rotenone treatment.

We recognize that the locomotor tests employed are not comprehensive but believe that they are, indeed, adequate to demonstrate rotenone-treated animals in this study have the same locomotor abnormalities as those reported previously. The cylinder test (rearing test) and open-field test are well established (Hua et al., 2002; Rial et al., 2014; Schaar et al., 2010; Su et al., 2018), and verified the locomotor abnormalities known in this model.

### Perspective

We believe that the rat rotenone model is valuable for further neurochemistry-oriented research testing and applying the catecholaldehyde hypothesis regarding the pathogenesis and treatment of PD. Several directions can be taken. The rotenone model provides a platform for further studies on *in vivo* mechanisms by which DOPAL interacts with alpha-synuclein and other intracellular proteins (Jinsmaa et al., 2018) to threaten neuronal homeostasis. The catecholamine functional abnormalities reported here render the model suitable for testing interventional strategies that might salvage dysfunctional catecholaminergic neurons. This understanding might lead to novel disease-modifying treatments, such as enhancing VMAT or ALDH activity (Lohr and Miller, 2014; Xiong et al., 2016; Chiu et al., 2015), or to combined strategies for which this model is useful. We believe that, in the future, combination pharmacotherapy will prove to be effective for neurodegenerative diseases just as in other areas of medicine.

In conclusion, the rat rotenone model exhibits a set of functional abnormalities in catecholaminergic neurons that reproduces the pattern found in PD putamen. These include a vesicular storage defect, decreased ALDH activity and DOPAL build-up. The catecholaminergic functional abnormalities reported here render the rat rotenone model a suitable *in vivo* platform for studying the catecholaldehyde paradigm for the neurodegenerative process in PD.

## MATERIALS AND METHODS

The animal research procedures in this study complied with the guidelines of and were approved by the Animal Care Committee of the Chaim Sheba Medical Center, Tel Hashomer, Israel, where the animal experiments took place. The neurochemical assays were done in the laboratory of the Autonomic Medicine Section in the Division of Intramural Research of the National Institute of Neurological Disorders and Stroke at the National Institutes of Health, Bethesda, MA, USA.

### Animals

Sprague-Dawley male rats (200±20 g, 10 weeks old) were obtained from Harlan Laboratories (Jerusalem, Israel). The rats were acclimated for at least 3 days before surgery (minipump implantation) and experiments. Postoperatively, the rats were housed separately in cages in an animal care facility at 22°C with a 14-h light (6:00–20:00) and 10-h dark (20:00–6:00) cycle, with free access to food and water.

### Materials

Dimethyl sulfoxide (DMSO) and polyethylene glycol (PEG) were mixed in a 1:1 ratio and used as the vehicle for rotenone. Rotenone was emulsified in a 5.6 mg/ml of the DMSO/PEG mixture and injected into an Alzet minipump until the pump was full. For vehicle-treated rats, the 1:1 DMSO/PEG mixture alone was used. Pumps were prepared to release rotenone at a rate of 2 mg/kg/day. This dose was selected after a pilot experiment to determine the optimal dosage regimen.

### Study protocol

For minipump insertion, each rat was anesthetized using 3% isoflurane and placed in a small animal stereotaxic frame. An Alzet minipump was implanted subcutaneously at the dorsum of the neck. In this model, the parkinsonian behavioral phenotype is evident after several days of treatment (Cannon et al., 2009); therefore, the duration of the study was 10 days. Rats were stratified into two groups: vehicle-treated (Group 1, *n*=8), rotenone-treated (Group 2, *n*=11). In Group 1 the minipump contained only the vehicle, in Group 2 the minipump contained rotenone emulsified in the vehicle. On day 10, animals were euthanized using an overdose of 3% isoflurane. Tissue harvesting began immediately after sacrifice. Brains were removed and dissected, striata explored and central portions sampled. Tissue the size of ∼3×3×3 mm was taken and immediately placed on ice, transferred to a plastic cryotube, frozen in liquid nitrogen and stored at −80°C or in dry ice until assayed.

### Motor function tests

Motor function tests were carried out on days 0, 1 and 9. To measure rearing behavior and spontaneous exploration a modified cylinder test (Hua et al., 2002; Schaar et al., 2010) was used, and rats were placed individually in a rectangular plastic box of 30×34×16 cm (length×width×height). Each rat was allowed free exploration within the apparatus while being videotaped for 2 min. Total rearing number was the number of times the animal was leaning on its hind legs and dethatching its forelegs*.* Change in activity time was the time in seconds during which the animal was moving at baseline minus the total time the animal was moving on day 1 or 9.

### Neurochemical assays

Neurochemical assays were carried out by personnel blinded regarding the treatment group until assay results were recorded. Freshly thawed tissue from striatum was homogenized in a mix of 0.2 M phosphoric acid and 0.2 M acetic acid (20/80%) under a fume hood. Aliquots of the supernatants were assayed for catechol contents using high-performance liquid chromatography (HPLC) as described previously (Goldstein et al., 2013). Briefly, the supernatant subjected to aluminium oxide extraction followed by reverse phase, ion-pairing liquid chromatography with series electrochemical detection. There were three electrodes in series, with the first set at an oxidizing potential and the third at a reducing potential. Signals from the third potential were recorded, providing a measure of reversibly oxidized species. Norepinephrine, dopamine, DOPAC, DHPG, DOPAL and DOPA were assayed simultaneously, and their concentration in tissue was expressed in pmol/mg (wet weight).

### Statistics

Motor function data were analyzed using factorial analysis of variance with Tukey's post-hoc test. To compare results of biochemical analyses between the groups, independent-means *t*-tests were conducted on log-transformed data. Data are provided as the mean±s.e.m. Statistical significance was defined to be *P*<0.05.
